# Serum Progranulin as a Potential Diagnostic Predictor in Neonatal Sepsis

**DOI:** 10.7759/cureus.88360

**Published:** 2025-07-20

**Authors:** Dandan Bei, Yan Cheng, Ruoxuan Jiang, Huafeng Wang

**Affiliations:** 1 Department of Neonatology, The Second Hospital of Anhui Medical University, Hefei, CHN

**Keywords:** biological marker, neonatal sepsis, nlr, progranulin, wbc

## Abstract

Background: The study aimed to evaluate the diagnostic and prognostic power of progranulin (PGRN) in neonatal sepsis.

Methods: We enrolled 67 newborns hospitalized for sepsis or suspected infection. Serum biomarkers, including PGRN, were quantified at admission. The relationship between PGRN levels and sepsis severity, hospital stay duration, and diagnostic accuracy was analyzed.

Results: Neonates with sepsis exhibited elevated PGRN concentrations, which correlated directly with prolonged hospitalization. PGRN emerged as a robust prognostic biomarker for neonatal sepsis, indicating the highest predictive power (AUC: 0.956; 95% CI: 0.875-0.991; *P* < 0.001). At a threshold of 80.4 ng/mL, PGRN achieved 90.91% sensitivity, 80.3% specificity, and an AUC of 0.760.

Conclusion: This study indicated that PGRN may be an excellent biomarker for early diagnosis and guiding clinical management of neonatal sepsis.

## Introduction

Neonatal sepsis remains a leading cause of hospitalization and mortality in low- and middle-income countries (LMICs), representing a significant global health burden despite progress in maternal and newborn care [1]. In China, neonatal sepsis, a complex clinical syndrome characterized by severe infections, caused by various pathogenic organisms, such as gram-negative bacilli, is prevalent, with extremely high mortality rates among affected infants ranging from 13.2% to 46% [2]. Early symptoms of neonatal sepsis are not specific and can be easily overlooked by parents or healthcare professionals, leading to misdiagnosis [2]. As a result, rapid and effective diagnostic methods are necessary to reduce neonatal mortality.

Though blood culture remains the gold standard not only for distinguishing infection but also for providing critical insights into antibiotic sensitivity, it requires 48-72 hours to identify pathogens, with a positive rate ranging from 8% to 73% [3]. Furthermore, its positive yield is extremely influenced by several factors, including the timing of sample collection, collection techniques, and culture medium quality, which limits its clinical utility [4]. C-reactive protein (CRP) and procalcitonin (PCT) have emerged as valuable biomarkers for recognizing infectious inflammation and are widely utilized in sepsis diagnosis. However, its low specificity and hysteresis limited its application in neonatal sepsis [5]. Recent studies have demonstrated that novel biomarkers, such as resistin, haptoglobin, and sTREM-1, could be applied for clinical prognostic identification of neonatal sepsis, but their expensive and time-consuming disadvantage hinders widespread implication in neonatal sepsis [6,7]. 

The neutrophil-to-lymphocyte ratio (NLR) and white cell counts (WBC), derived from routine blood cell counts, could help recognize sepsis and initiate treatment while awaiting culture results [8]. Previous studies have suggested that NLR and WBC can be used for identifying early-onset neonatal sepsis [9]; however, their reliability remains unclear.

Emerging evidence highlights progranulin (PGRN), an anti-inflammatory cytokine expressed in epithelial and immune cells, which regulates cell proliferation, tissue repair, and cancer development. PGRN plays an important role in the development of acute lung injury and the progression of pneumonia, and it may serve as a prognostic biomarker for sepsis severity in adults, with elevated levels correlating with adverse outcomes [10,11]. However, its role in neonatal sepsis is poorly understood. This study aims to evaluate serum PGRN, WBC, and NLR as early diagnostic and prognostic tools for neonatal sepsis.

## Materials and methods

Study design and settings

A case-control study was performed at a single medical institution to assess the value of PGRN, NLR, and WBC as diagnostic indicators for neonatal sepsis. A total of 67 newborns were enrolled, who were admitted to the neonatal clinical unit of The Second Hospital of Anhui Medical University, Hefei, between January 1, 2021, and December 1, 2024, with suspected sepsis. The eligibility criterion for neonatal sepsis is described according to the European Medicines Agency report on the Expert Meeting on Neonatal and Pediatric Sepsis [12,13]. Neonatal sepsis was defined as the patient exhibiting evidence of infection confirmed by positive culture, and at least two clinical signs and two laboratory abnormalities regarding clinical, hemodynamic status, tissue perfusion, or inflammatory variables related to inflammation, and for whom there was a non-infectious explanation for the observed clinical and laboratory manifestations. Suspected sepsis was defined by the abnormalities of the listed clinical or laboratory manifestations as follows: (1) Abnormal temperature: (i) core temperature ＞38.5°C or ＜36°C and/or (ii) temperature instability; (2) Respiratory dysfunction: (i) apnoea, (ii) tacypnoea, (iii) requirement for ventilation support; (3) Skin and subcutaneous lesions: (i) petechial rash, (ii) sclerema; (4) Cardiovascular abnormalities: (i) bradycardia, (ii) tachycardia, (iii) rhythm instability, (iv) diminished urinary output (<1 ml/kg/h), (v) hypotension, (vi) mottled skin, (vii) impaired peripheral perfusion; (5) Gastrointestinal: (i) feeding intolerance, (ii) inadequate sucking reflex, (iii) abdominal distention; (6) Non-specific indicators: (i) irritability, (ii) lethargy, (iii) hypotonia. The control group was defined as newborns requiring medical intervention but without evidence of infection (e.g., physiological jaundice, feeding intolerance), who were admitted to the neonatal intensive care unit (NICU) during the same period. All informed consents were obtained from legal guardians. Participants were divided into three groups: sepsis-positive (culture positive), suspected sepsis (culture negative), and control. Inclusion criteria for the group of cases were as follows: (1) newborns admitted to the NICU during the study period; (2) culture positive; (3) culture negative but clinical diagnosis of sepsis; (4) complete laboratory analysis data. Exclusion criteria were as follows: (1) non-obvious consent to use data from the legal representative; (2) missing data; (3) presence of subchorionic hemorrhage during pregnancy. The study protocol was approved by the Ethics Committee, The Second Hospital of Anhui Medical University (Approval No. YX2024-143).

Data collection

The clinical parameters, including gestational age (GA) at birth, birth weight (grams), sex, delivery mode (cesarean or vaginal), pregnancy complications (e.g., intrauterine growth restriction, maternal hypertension, gestational diabetes, preterm premature rupture of membranes, preterm birth), 1-minute Apgar score, parity, and positive cervical culture, were collected from the neonate's digital medical records. At admission, venous blood samples were taken from every newborn for laboratory testing. The blood culture that came positive was subcultured on 5% human blood agar and nutrient agar. Further isolation and antibiotic susceptibility were performed using the disc diffusion method. The laboratory results, such as blood culture results, complete blood count (CBC), and CRP, were also collected by physicians. Blood culture yielding a positive result was subcultured on 5% human blood agar and nutrient agar. Subsequently, bacterial isolation and antimicrobial susceptibility testing were conducted via the disk diffusion method. The levels of PGRN in the peripheral venous blood were measured using enzyme-linked immunoassay kits (Human Progranulin ELISA Kit - Quantikine, R&D Systems, Minneapolis, MN, USA) following the manufacturer’s instructions.

Statistical analysis

Statistical analyses were conducted using IBM SPSS Statistics for Windows, version 23.0 (IBM Corp., Armonk, NY, USA) and MedCalc, version 19.4.0 (MedCalc Software Ltd, Ostend, Belgium). Data were presented as median (interquartile range, IQR) or frequency (percentage). Mann-Whitney U test or Kruskal-Wallis ANOVA for non-normally distributed continuous variables, and Χ^2^ test for categorical variables were performed to analyze group differences. The area under the curve of the receiver operating characteristic curve (ROC-AUC) was used to determine the predictive values of different variables by performing the ROC curve, and *P*<0.05 was considered to be statistically significant.

## Results

Baseline characteristics

This study enrolled 67 infants, including 22 (32.8%) with confirmed sepsis and 45 (67.2%) without. Among septic neonates, 17 (77.3%) exhibited early-onset sepsis (EOS), while 5 (22.7%) had late-onset sepsis (LOS). Out of 67 newborns, 41 (61.1%) were males and 26 (38.9%) were females. No significant differences were observed in infant sex, cesarean birth, fetal growth restriction, and APGAR score at 1 min between septic and non-septic infants. Similar results have also been observed in preterm birth, fetal anemia, gestational age, and infant weight at birth. However, the prevalence of cesarean delivery was significantly higher in septic neonates compared to those without sepsis (39.5% vs. 20.0%; *P* < 0.05) (Table 1). Preterm birth and fetal growth restriction were more frequent in the septic group, but lacked statistical significance.

**Table 1:** Baseline characteristics NLR: neutrophil-to-lymphocyte ratio; PGRN: progranulin

Characteristics	Control	Infection	Sepsis
Gender (male)	21 (16)	24 (11)	22 (14)
Infant weight (grams)	3268 ± 541.1	3447 ± 456.4	3116 ± 577
Cesarean birth	11	6	10
Fetal growth restriction	1	11	14
Apgar score at 1 min	10	10	10
Neonatal anemia (Hb < 13.5 g/dL)	1	2	7
Neonatal hemoglobin	166.1 ± 18.48	174.1 ± 17.66	150.6 ±2 8.08
Period of gestation in days	272.7 ± 6.1	278.3 ± 6.5	267.2 ± 28.55
Onset of sepsis (EOS)	1	23	17
Hospitalization period	4.38 ± 1.11	9.38 ± 4.39	18 ± 9.36
NLR	0.54 ± 0.11	4.29 ± 1.37	7.98 ± 8.94
WBC (cells/mm³)	10.61±2.32	17.72±5.65	24.67 ± 12.4
Platelet (*10^9^/L)	350.2±77.4	244.2±43.6	265.9±90.43
PGRN (ng/ml)	46.51 (39.26–53.78)	75.48 (69.85–82.02)	111.8 (91.56–131.1)
Maternal characteristics			
Parity	5	10	10
Pregnancy-induced hypertension	3	0	7
Gestational diabetes	5	7	9
Urinary tract infections	0	1	1
Premature rupture of membranes	6	3	6
Positive cervical culture	0	0	0

**Figure 1:**
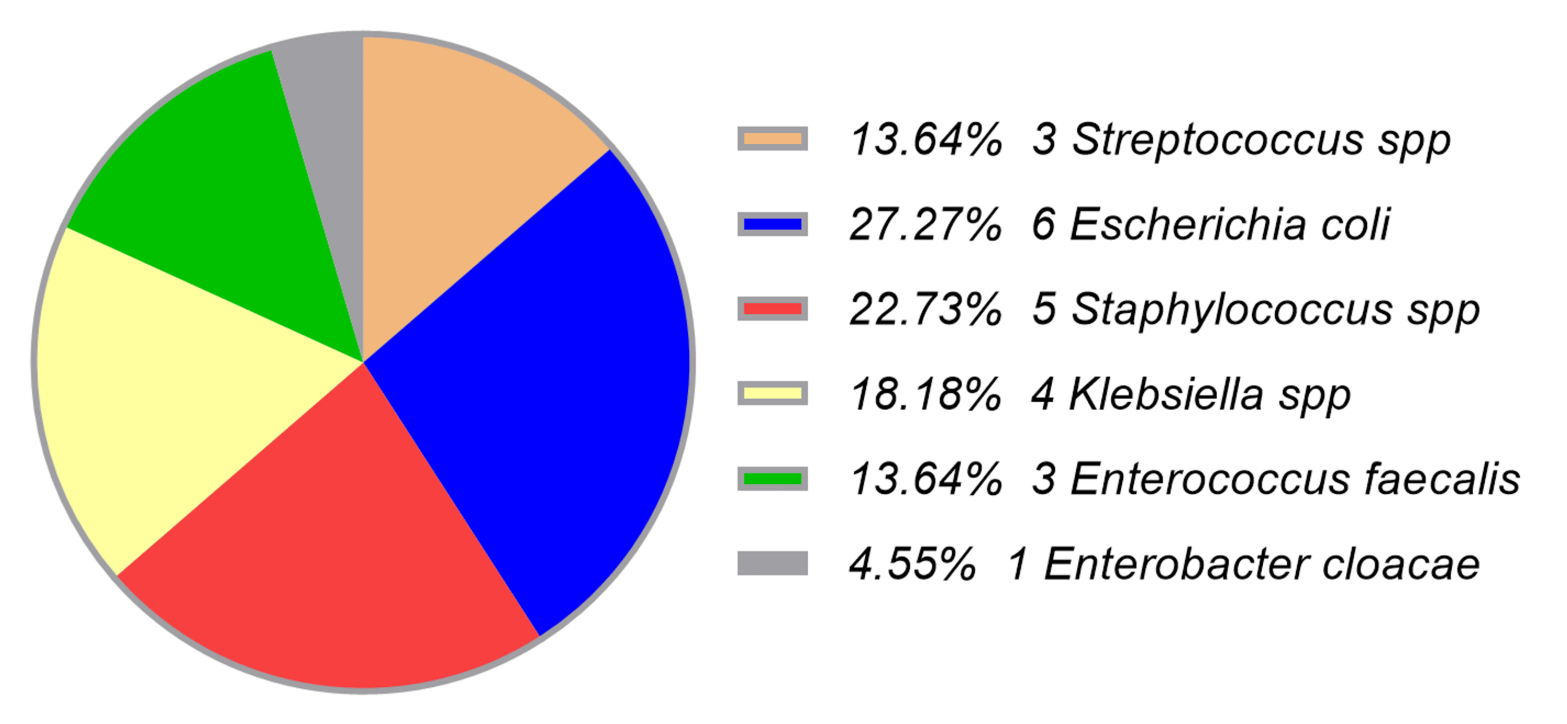
Pathogens isolated from blood culture

The most frequently isolated pathogen from blood cultures was *Escherichia coli*. Others included *Streptococcus* spp., *Staphylococcus* spp., *Klebsiella* spp., *Enterococcus faecalis*, and *Enterobacter cloacae* (Figure 1).

Levels of PGRN in different groups and their predictive value for neonatal sepsis

**Figure 2:**
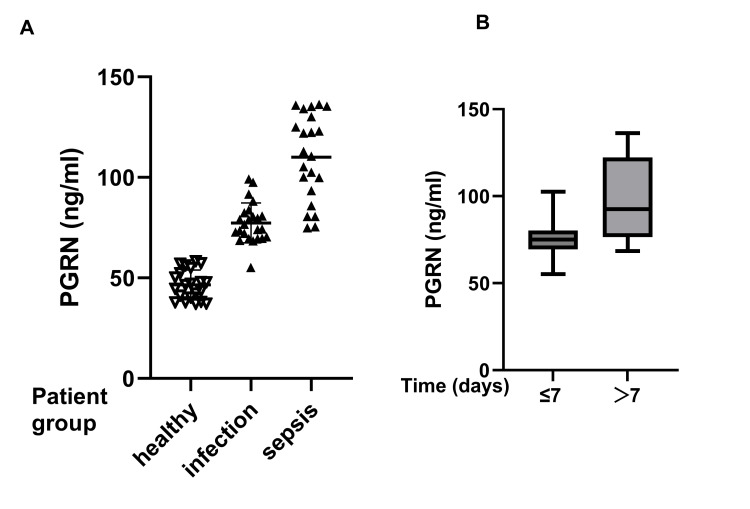
Levels of PGRN in different groups (A) Levels of PGRN in the control, infection, and sepsis groups; (B) Association of the levels of PGRN with duration of hospital stay (days) PGRN: progranulin

Elevated plasma levels of PGRN were observed in different groups, with values of 46.51 ng/ml (IQR: 39.26-53.78) in control groups, 75.48 ng/ml (IQR: 69.85-82.02) in infection groups, and 111.8 ng/ml (IQR: 91.56-131.1) in sepsis neonates. Further, the levels of septic neonates were significantly higher than those of infectious patients or the controls. The levels of serum PGRN correlated with hospital stay duration (≤7 days vs. >7 days): shorter stays had a lower median PGRN (75.12 ng/mL; IQR: 69.43-80.27) versus prolonged stays (92.55 ng/mL; IQR: 76.49-122.3) (Figure 2). The wider range in the latter group suggests the variability of serum PGRN levels may reflect disease severity or prolonged hospitalization. Consistent with PGRN, WBC counts and NLR were also elevated in septic neonates compared to infected or control groups (*P *< 0.05).

**Figure 3:**
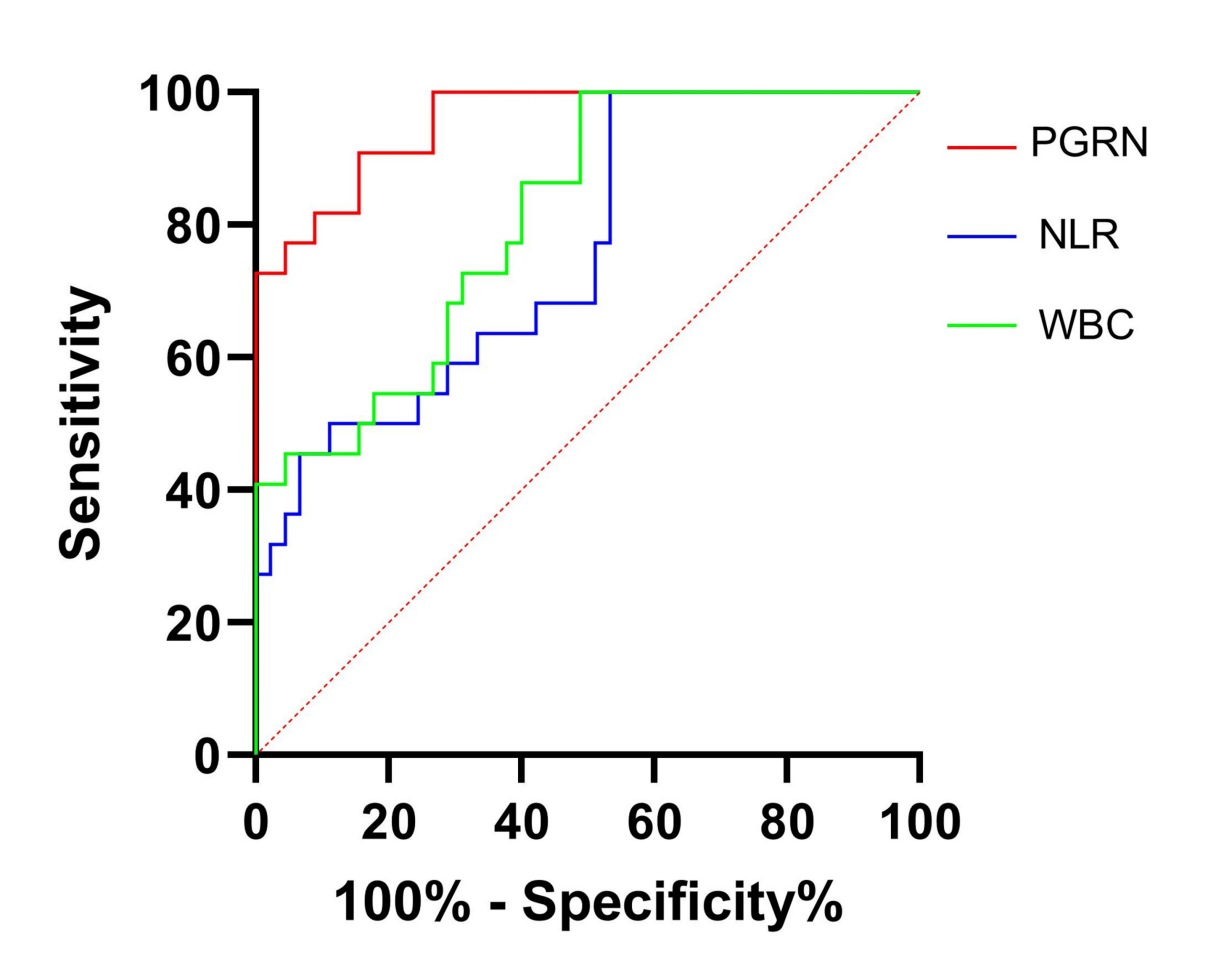
ROC analysis of PGRN, NLR, and WBC in predicting neonate sepsis ROC: receiver operating characteristic curve; PGRN: progranulin; NLR: neutrophil-to-lymphocyte ratio

The ROC analysis showed PGRN’s robust diagnostic power for neonatal sepsis (AUC: 0.956; 95% CI: 0.875-0.991; P < 0.001) (Figure 3). At a threshold of 80.4 ng/mL, PGRN exhibited 90.91% sensitivity (95% CI: 70.8-98.9) and 84.44% specificity (95% CI: 70.5-93.5), with a positive likelihood ratio of 5.84 (95% CI: 2.9-11.7) and a negative likelihood ratio of 0.11 (95% CI: 0.03-0.4), respectively. The conventional WBC counts and NLR also were calculated, and the results showed a sensitivity of 100% (95% CI: 84.6-100.0) and 100% (95% CI: 84.6-100.0), a specificity of 51.11% (95% CI: 35.8-66.3) and 46.67% (95% CI: 31.7-62.1), and an AUC of 0.81 (95% CI: 0.696-0.896) and 0.76 (95% CI: 0.640-0.856), respectively (Figure 3).

## Discussion

Neonatal sepsis remains a critical challenge in pediatric healthcare, necessitating early diagnosis and treatment to improve survival outcomes of neonatal sepsis. Serum PGRN, an adipokine associated with inflammation, has recently gained interest for its prognostic value for mortality in septic conditions. This study investigated the expression of serum PGRN levels in newborns with infection or sepsis, aiming to explore its ability for early diagnostic potential for neonatal sepsis, which is of great value for making critical decisions in neonatal infection care.

In this study, our findings demonstrated that serum PGRN, WBCs, and NLR as significant predictive biomarkers for neonatal sepsis, with PGRN showing the highest predictive power (AUC: 0.956 (95% CI: 0.875 to 0.991); P < 0.001). At a threshold of 80.4 pg/mL, PGRN exhibited a sensitivity of 90.91%, specificity of 80.3%, and an AUC of 0.760. Notably, serum PGRN levels significantly increased in early-onset sepsis (EOS) cases compared to late-onset sepsis, correlating with the predictive power. Multivariate regression analysis demonstrated WBC and NLR had a sensitivity of 100% and 100%, a specificity of 51.11% and 46.67%, and an AUC of 0.81 and 0.76, respectively. Many biomarkers, such as CRP and PCT, are widely used for infection diagnosis and monitoring in clinical practice. Nevertheless, CRP is unsuitable for early screening and prognosis prediction of sepsis due to its delayed elevation during inflammation [14]. Increased serum PCT concentrations are only observed in bacterial-induced sepsis, not in viral infections. However, PCT levels are also altered in patients with severe renal failure, rendering it suboptimal as a sepsis assessment marker. PGRN concentrations remain relatively stable during the initial phase of sepsis, and the optimal PGRN cut-offs may not require adjustment for neonatal sepsis diagnosis. Furthermore, high levels of PGRN perform better sensitivity to differentiate infected and uninfected neonates at 0-24 h and 25-48 h. In contrast, CRP and PCT levels exhibit substantial short-term fluctuations, diminishing their utility for early detection and dynamic evaluation of disease severity [15]. PGRN seemed to be more effective than conventional parameters (SOFA scores and APACHE II scores) in predicting the prognosis of sepsis [10]. Thus, PGRN appears to be an excellent biomarker for screening EOS compared to conventional markers. Moreover, the combination of PGRN and WBC count further enhanced diagnostic accuracy compared to either marker alone.

Consistent with our results, elevated PGRN levels have been observed in adult and EOS sepsis cohorts relative to the respective controls [16,17]. Clinical studies indicated that high PGRN levels contributed to the development and prognosis of sepsis [16], while preclinical studies further highlight its role in suppressing the secretion of lipopolysaccharide (LPS)-induced inflammatory cytokines and chemokines, indicating its protective potential against sepsis [18]. However, PGRN aggravates lethal *Candida albicans* sepsis by exacerbating the inflammatory response and antifungal immunity [19], indicating its temporal and tissue-specific roles in inflammatory diseases. As a cysteine-rich secretory protein (CRISP), PGRN is a pivotal regulator in immunity and inflammation responses [20, 21]. Immune cells, including macrophages, dendritic cells, and alveolar epithelial cells, secrete and release PGRN into the bloodstream during sepsis, which can be regarded as a hallmark pathological feature [22]. Mechanistically, PGRN could inhibit inflammatory cytokine and chemokine synthesis by enhancing C/EBPα-regulated IL-10 transcription while blocking ubiquitin ligase/proteasome-mediated protein degradation [23,24]. Additionally, PGRN also improved the survival rate by suppressing the apoptosis in sepsis-induced acute lung injury [25]. These dual roles position PGRN as a therapeutic candidate, though its molecular mechanisms deserve further exploration.

The NLR, which reflects systemic inflammation and neutrophil-lymphocyte balance, is particularly relevant in gram-negative bacteria-induced sepsis. Prior research explores its potential as an early diagnostic biomarker for neonatal sepsis [26]. In addition, NLR has been associated with disease prognosis and survival rates in diseases with systemic inflammation, particularly in some bacterial and bloodstream infections [27,28]. Similarly, WBC counts, though integral to inflammation and host defense, exhibit limited specificity in isolation. Thus, integrating WBC with other biomarkers (e.g., PGRN, NLR) is essential for early diagnostic precision, meaning the need for multidimensional clinical assessments plays a crucial role in inflammation and host defense. Therefore, WBC should be interpreted in conjunction with other biomarkers to enhance diagnostic accuracy, and clinical decision-making should be grounded in a multidimensional assessment, avoiding overreliance on any single parameter [29,30].

Strengths and limitations

The present study is subject to several limitations. Firstly, its retrospective design and small patient cohort at a single institution may lead to some overestimation in classification and differentiation in early- versus late-onset sepsis. Secondly, the predictive model is derived from a single-center study utilizing internal validation, raising concerns about generalizability. Lastly, categorizing neonates based solely on blood culture results has inherent limitations, owing to the sensitivity and specificity of blood cultures. Despite these limitations, our study provides evidence for the usefulness of PGRN and NLR in the diagnosis of neonatal sepsis. Future studies with larger samples, multiple centers, and more comprehensive parameters will undoubtedly shed more light on the significance of PGRN and NLR in managing neonatal sepsis.

## Conclusions

Based on the study results, it can be concluded that there is a significant correlation between progranulin and NLR and neonatal sepsis. PGRN was an excellent predictor for EOS and hospital stay duration in neonatal sepsis, and PGRN exhibited the highest predictive power for screening neonatal sepsis. PGRN was a reliable marker for early diagnosis and guiding clinical management of neonatal sepsis.
